# Q Fever Endocarditis in HIV-Infected Patient

**DOI:** 10.3201/eid1003.030971

**Published:** 2004-03

**Authors:** Miguel G. Madariaga, Joseph Pulvirenti, Marin Sekosan, Christopher D. Paddock, Sherif R. Zaki

**Affiliations:** * Cook County Hospital, Rush Medical College, Chicago, Illinois, USA; † Centers for Disease Control and Prevention, Atlanta, Georgia, USA

**Keywords:** Q fever, Coxiella burnetii, HIV, endocarditis

## Abstract

We describe a case of Q fever endocarditis in an HIV-infected patient. The case was treated successfully with valvular replacement and a combination of doxycycline and hydroxychloroquine. We review the current literature on Q fever endocarditis, with an emphasis on the co-infection of HIV and *Coxiella burnetii*.

*Coxiella burnetii* is classified in the order Legionellales and is closely related to *Legionella* and *Francisella* spp (*1*). This zoonotic agent has been isolated from various birds, mammals, and arthropods and is considered endemic in cattle in some regions of the United States (*2*,*3*). Although usually nonpathogenic in animals, outbreaks of *C. burnetii*–induced abortions have been described in goats and sheep. In humans, *C. burnetii* is acquired primarily by inhaling infectious dust (*4*). The bacteria are able to survive in a sporelike form under harsh environmental conditions and are extremely contagious: a single organism can cause disease (*5*). Q fever manifests as acute or chronic disease. The acute disease may include an undifferentiated febrile syndrome, pneumonia, or hepatitis. The most common chronic symptom is endocarditis (*6*). We describe a case of Q fever endocarditis in a patient with HIV infection. This co-infection has never been reported in the United States and rarely has been described elsewhere.

## The Study

A 46-year-old man with HIV infection was admitted to an emergency department in August 2002 with sudden onset of chest pain and shortness of breath. The patient was born in Mexico and recalled contact with farm animals and consuming unpasteurized dairy products while raised in the state of Chihuahua. He migrated to United States in 1987 and returned periodically to Mexico, most recently in 1998. While residing in the United States, the patient had no known direct or indirect exposures to ruminants.

Ten months before admission (November 2001), the patient had been hospitalized with pneumonia and was diagnosed with HIV infection. One month later, the patient was hospitalized because of *Candida* esophagitis and thrombocytopenia. A bone marrow biopsy obtained during that hospitalization showed adequate megakaryocytes, mild megaloblastic changes in erythroid precursors, and adequate iron stores. Special stains and cultures for acid-fast bacilli and fungi were negative, as were blood cultures for *Mycobacterium avium* complex. During that admission a diastolic murmur was noted, and an echocardiogram showed severe aortic insufficiency with a thickened aortic valve. The patient was discharged on antiretroviral therapy with lamivudine, stavudine, and nelfinavir, diuretics and ACE inhibitors.

Seven weeks before the first hospitalization in November 2001, the patient was admitted with headache, neck rigidity, and chills. Computed tomography scans of the head and neck and a lumbar puncture showed no abnormalities. The symptoms resolved with the use of empiric intravenous vancomycin and ceftriaxone. Because of persistent thrombocytopenia, a second bone marrow biopsy was performed in February 2002. Granulomas were identified in the biopsy, although special stains for acid-fast bacilli and fungi were negative. Treatment with clarithromycin, ethambutol, and rifabutin for presumed *M. avium* complex was initiated. and the patient was discharged.

On examination, pulse was 101 beats per minute, temperature was 37.0°C, respiratory rate was 22 beats per minute, and blood pressure was 113/42 mm Hg. Mild temporal wasting, absence of oral thrush, poor dentition, positive jugular venous distention, bilateral crackles in the lungs, a decrescendo diastolic murmur in the left sternal border, “water hammer” pulses, and clubbing of the digits were found. Serum sodium level was 129 mEq/L; creatinine 1.4 mg/dL; albumin 3.2 mg/dL; normal liver function tests; leukocyte count 8,800/μL, with 90.8% neutrophils and 7.3% lymphocytes and hemoglobin 8.2 g/dL and platelets 72,000/μL. Antibodies to nuclei and smooth muscle were detected at titers of 1:40 and 1:160, respectively. CD4^+^ lymphocyte count was 82 cells/mm^3^ and viral load 2,838 copies/mm^3^. Chest x-ray showed cardiomegaly and congestive heart failure. An electrocardiogram showed a left anterior hemiblock and a first-degree atrioventricular block. A transthoracic echocardiogram showed a large vegetation on the aortic valve and severe aortic insufficiency. Other findings included left ventricular dysfunction, a structurally normal mitral valve with a small vegetation on the atrial surface of the anterior leaflet, and mild mitral regurgitation. Empiric treatment for bacterial endocarditis was initiated with oxacillin, gentamicin, and ampicillin. Because of severe aortic insufficiency, the patient’s aortic valve was surgically resected and showed a severely fenestrated, tri-leaflet valve with bulky, white, irregular vegetations. The mitral valve was free of vegetations, but was perforated and required repair with an autologous pericardial patch. Initial histopathologic evaluation of the excised aortic valve reported scant inflammation and extensive calcification and hyalinization. The patient did well postoperatively and was discharged after 4 weeks of intravenous antimicrobial therapy. Blood cultures obtained before antimicrobial drugs were administered failed to grow routine aerobic or anaerobic bacteria, mycobacteria, or fungi.

After discharge, serum samples tested by an indirect immunofluorescence antibody (IFA) assay showed immunoglobulin (Ig) G antibodies reactive with phase I and II antigens of *C. burnetii* at reciprocal titers of 16,384 and 16,384, respectively. The resected valve was sent to the Centers for Disease Control and Prevention. Histopathologic evaluation showed calcification and hyalinization and foci of prominent mononuclear infiltrates with occasional multinucleated giant cells. An immunohistochemical stain for *C. burnetii* using a polyclonal mouse primary antibody reactive with *C. burnetii* was applied to sections of valve tissue. Abundant intracellular staining of *Coxiella* antigens was identified in foamy macrophages in areas of inflammation and calcification (Figure). The patient was started on doxycycline 100 mg per os each day and hydroxychloroquine 400 mg per os each day. During a follow-up visit 4 months after hospitalization, the patient was clinically asymptomatic, platelet count was 146/μL, albumin was 4.2 mg/dL, and no antinuclear antibodies were detected. Follow-up antibody titers against phase I and II antigens of *Coxiella* were both 4,096.

**Figure:**
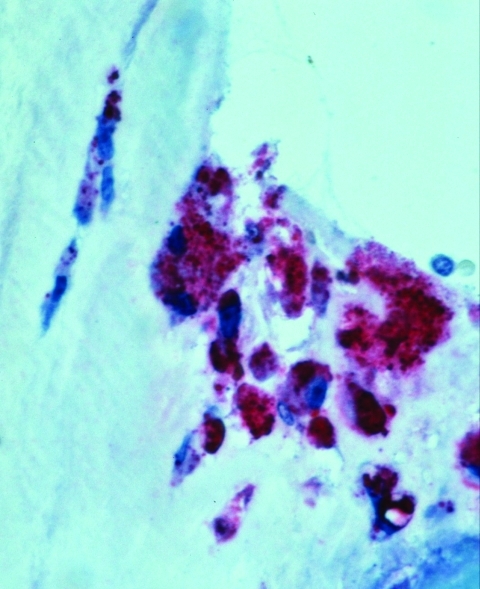
Immunohistochemical localization of *Coxiella burnetii* antigens in the aortic valve of a patient co-infected with HIV. Intact bacteria and fragment antigens are identified predominantly within macrophages in the fibrosed and calcified valve tissue. (Immunoalkaline phosphatase stain with naphthol phosphate fast-red substrate and hematoxylin counterstain, original magnification X100).

## Conclusions

Q fever has a worldwide distribution (*7*) and, in the United States, was made nationally notifiable in 2000. The disease is not reportable in Mexico, our patient’s country of origin, and only one case report of Q fever endocarditis has been published from that country (*8*).

Pneumonia developed in our patient several months before endocarditis was diagnosed; however, the role of *C. burnetii* as the etiologic agent of his prior pulmonary disease is not known. A syndrome characterized by absence of fever; insidious aortic valvular dysfunction, leading to congestive heart failure; anemia; thrombocytopenia; and autoantibodies subsequently developed in our patient. Routine blood cultures were negative. Culture-negative endocarditis is implicated in 6% of cases of infective endocarditis in HIV-infected patients. Some of the pathogens identified as the cause of culture-negative endocarditis in this patient cohort include *Bartonella*, *Coxiella,* and *Arcanobacterium*. However, because diagnostic assays for Q fever are attempted infrequently, the number of culture-negative endocarditis cases caused by *C. burnetii* is unknown (*9*–*11*).

Although infections with certain intracellular organisms (e.g., *Mycobacterium*, *Salmonella*, and *Leishmania* spp.) are more frequent and have more severe signs and symptoms in HIV-infected patients, the prevalence and severity of Q fever in persons infected with HIV compared with the general population remain controversial. Raoult et al. (*12*) found a threefold increase in prevalence of Q fever serologic findings among HIV-infected patients in Marseille, France (2.4% vs. 0.8%), and determined an annual incidence of 2.7 per 100,000 in the general population and 33 per 100,000 in HIV-seropositive patients (*4*). However, other seroprevalence studies failed to confirm an increase of Q fever in HIV-seropositive patients (*13*–*15*). A study from Italy described two outbreaks of Q fever in a residential facility for drug abusers. In the first epidemic, the Q fever attack rate was 45.9% for HIV-seropositive persons compared with 25.1% for HIV-seronegative persons. However, during the second outbreak 1 year later, no significant association with HIV coinfection or CD4^+^ cell count existed, and no significant differences occurred in the levels of antibodies or the clinical signs and symptoms between these patient cohorts in either outbreak (*16*).

Even less information is available about Q fever endocarditis in persons infected with HIV. *C. burnetii* is controlled by a nonsterile immunity and perhaps is never cleared from an infected patient (*17*). Because *C. burnetii* is an intracellular pathogen, Q fever endocarditis might be expected to occur with greater frequency in HIV-infected patients than in the general population; however, most cases of chronic Q fever occurring in immunosuppressed patients have been reported among persons with cancer (*6*) and only rarely among HIV-infected persons (*6*,*12*).

Q fever endocarditis was suspected in our patient on the basis of his clinical manifestations, history of exposure to farm animals, and absence of bacterial growth in routine blood cultures. Conventional blood cultures for *C. burnetii* are characteristically negative, but use of shell vial cell culture assay techniques are more sensitive and less hazardous than conventional blood cultures (*18*).

The diagnosis in our patient was confirmed by serologic and immunohistochemical methods. Chronic Q fever can be diagnosed by detection of high anti-phase I IgG antibody titers by IFA, complement fixation, or enzyme immunoassay (*19*). IFA is considered the serologic standard criterion and was the test used in our patient. Nonspecific, low-dilution seropositivity for *Coxiella* has been reported in HIV-seropositive persons by IFA (12), but our patient had very high titers compatible with *Coxiella* infection. Also, some patients with *C. burnetii* have been found to have false-positive results by HIV enzyme-linked immunosorbent assay (*20*,*21*). This finding was not the case in our patient, who had a confirmatory Western blot for HIV and a low CD4^+^ cell count.

Echocardiography showed a large vegetation in our patient. This finding is relatively unusual in patients with Q fever endocarditis, and transthoracic echocardiogram rarely demonstrates vegetation (*6*). Because of the difficulty of diagnosing Q fever endocarditis with the current Duke’s criteria, particularly when blood cultures are negative and vegetation is absent, a modification has been suggested so that a single positive blood culture or a high anti-phase I antibody titer is considered diagnostic (*22*).

A combination of doxycycline and hydroxychloroquine was given to our patient, who is currently clinically well. Although no data specifically describe the treatment of Q fever endocarditis in HIV-infected patients, the combination of both antimicrobial drugs appears to be an effective therapeutic regimen for this disease. Hydroxychloroquine increases the pH of phagolysosomes, enhancing the activity of doxycycline against *C. burnetii*. This combination of drugs can reduce the duration of treatment from 3–4 years to 18 months (*23*), although some authorities recommend that treatment be continued indefinitely.

Q fever endocarditis is a potentially severe infection, with a case-fatality ratio of approximately 24% in historical case series (*6*). Earlier diagnosis and newer treatment combinations may improve survival and decrease rates of recurrence. Further studies are required to evaluate the long-term prognosis of Q fever endocarditis in patients with HIV. Q fever is infrequently diagnosed in persons with endocarditis because of its relative rarity and because it is seldom considered in the differential diagnosis. However, it should be considered in all patients with culture-negative endocarditis, particularly those with appropriate risk factors that include past or current exposure to livestock.
